# Supplementary material for: Accuracy of Solid-State Residential Water Meters under Intermittent Flow Conditions
Source: Sensors (Basel). 2020 Sep 17;20(18):5339. doi: 10.3390/s20185339 (PMC7570974; doi:10.3390/s20185339)
Supplement: Supplementary file 1 [file sensors-20-05339-s001.pdf]

Supplementary material to

Accuracy of solid-state residential water meters under intermittent flow conditions

F. J. Arregui [1,*], L. Pastor-Jabaloyes [1], A. V. Mercedes [1] and F. J. Gavara [2]

[1] ITA-Grupo de Ingeniería y Tecnología del Agua, Dpto. de Ingeniería del Agua y Medio Ambiente, Universitat Politècnica de València, Camino de Vera s/n, 46022 València, Spain
[2] Head of metering department. FACSA, Calle Mayor 82-84, 12001 Castelló de la Plana, Spain
* Correspondence: farregui@ita.upv.es

Supplementary file overview

This file includes supplementary material associated with the research paper entitled '*Accuracy of solid-state residential water meters under intermittent flow conditions*' submitted to the Sensors journal. The file consists of two parts (S–A and S–B). The first part (S–A) provides supplementary material for Section 2 (Materials and Methods) of the research paper. This section presents statistical methods used in this study, and additional information in the form of graphs and tables on the test programme conducted. The second part (S–B) provides supplementary material for Section 3 (Results and discussion) of the research paper. For test T6 and T7, there is a comparison between the calculated and measured error. In addition, hypothesis testing methods are used to analyse the results obtained for M2 meters. As a note to the reader, this supplementary material accompanies and extends the information provided in the original research paper. Thus, the reading of both is recommended.

Table of contents

Supplementary file overview i

S-A. Supplementary material for (Section 2. Materials and Methods) 1

1. *Influence of meter resolution on test uncertainty* 1
2. *Number of repetitions conducted by test* 2
3. *Hypothesis testing methods overview* 5

S-B. Supplementary material for (Section 3. Results) 6

4. *Detection of typing errors in the database* 6
5. *Number of repetitions conducted by test* 7
6. *Results of test under inttermitent flow conditions per meter* 9
7. *Percentage of test results outside the error range of ±5%* 13
8. *Comparison of the calculated and measured error in tests T6 and T7* 15
9. *Hypothesis testing results* 19

References 28

List of tables

Table S-1. Number of repetitions conducted by test under steady flow conditions (T1). “Type of meter” and “Meter” are the first (*) and second (**) columns, respectively.. 2
Table S-2. Number of repetitions conducted by test under intermittent and constant flow conditions (T2, T3, T4, and T5). “Type of meter” and “Meter” are the first (*) and second (**) columns, respectively. 3
Table S-3. Number of repetitions conducted by test under intermittent and variable flow conditions (T6 and T7). “Type of meter” and “Meter” are the first (*) and second (**) column, respectively................................ 4
Table S-4. Average error obtained per test under steady flow conditions (T1) and meter. “Type of meter” and “Meter” are the first (*) and second (**) column, respectively. 7
Table S-5. Standard deviation obtained per test under steady flow conditions (T1) and meter. “Type of meter” and “Meter” are the first (*) and second (**) column, respectively. 8
Table S-6. Error calculated ($Errc$) and measured ($Errm$) by meter in T6 and T7 test. Electromagnetic and mechanical meters.. 16
Table S-7. Error calculated ($Errc$) and measured ($Errm$) by meter in T6 and T7 test. Ultrasonic meters.... 17
Table S-8. Descriptive statistics and results of the Shapiro-Wilk normality test (null hypothesis: the sample is normally distributed) of each defined group to compare the performance of M2 type meters of different ages. 20
Table S-9. Results of the hypothesis testing conducted to assess the difference between the performance of M2 meters manufactured in 2014 and 2017-2018. 23
Table S-10. Descriptive statistics and results of the Shapiro-Wilk normality test (null hypothesis: the sample is normally distributed) of each group established to compare the performance of M2 meters under steady and intermittent flow conditions. 24
Table S-11. Results of the hypothesis testing conducted to assess the difference between the performance of M2 meters under steady and intermittent flow conditions. 26

List of figures

Figure S-1. Error obtained for the M0016 meter in test T1 (steady flow conditions) at flow rates of 500, 1000, 1500, and 2000 L/h that has been repeated 14 times. 1
Figure S-2. Error of the sum of the volume recorded by the reference meters of each test line (Figure 2 of the research paper, MR004 and MR005) relative to the volume recorded by the reference meter at the output of the bench (Figure 2 of the research paper, MR003) used to conduct tests under intermittent flow conditions. 6
Figure S-3. Percentage of the total volume that passes through each line of the bench used to conduct tests under intermittent flow condition. 6
Figure S-4. Representation of the errors obtained in tests T2, T3, T4, T5, T6 and T7 by means of boxplots per meter, cyclic period, and test flow rate. Results in T1 (steady flow conditions) tests have been added as a reference. 12
Figure S-5. Percentage of tests results outside the error range of ±5%. DN20. 13
Figure S-6. Percentage of tests results outside the error range of ±5%. DN15. 14
Figure S-7. a) Error curve per meter (based on results of T1 tests). b) Consumption pattern P1 (lower flow rate range), P2 (higher variability) and P3 (upper flow rate range). 15

Figure S-8. Representation of the difference between the calculated and measured error per meter by means of boxplots, which are colored by type of meter. 18
Figure S-9. Quantile-quantile plot with marginal histograms to assess graphically the normality of the error distribution of the M2 (17-18) meters obtained in different tests 21
Figure S-10. Quantile-quantile plot with marginal histograms to assess graphically the normality of the error distribution of the M2 (14) meters obtained in different tests.......... 22
Figure S-11. Boxplots to compare the distribution of the error among the age groups. 23
Figure S-12. Two independent sample t test power calculations. Two sided tails and alpha of 0.05. *n* is de optimal sample size (number of units in each group for a balanced experiment design).......... 24
Figure S-13. Quantile-quantile plot with marginal histograms to graphically evaluate the normality of the error distribution of the M2 meters obtained in different tests. 25
Figure S-14. Comparison of error distribution of M2 meters obtained in tests under continuous and intermittent flow conditions through boxplots. 26
Figure S-15. Two paired sample t test power calculation. Two sided tails and alpha of 0.05. *n* is de optimal sample size (number of units in each group for a balanced experiment design).......... 27

S-A. Supplementary material for (Section 2. Materials and Methods)

1. *Influence of meter resolution on test uncertainty*

One of the assumptions of this study is to test solid-state meters in conditions as similar as possible to those found in the field. For this reason, the resolution of the meter, which is litres in all cases, was not modified in the laboratory. In order to determine how the uncertainty of the test was affected by this, up to 17 repetitions were conducted for some meters in certain tests under steady flow conditions (Table S-1). Figure S-1 shows quantile-quantile plots of the results obtained for the M0016 meter, which was tested 14 times at 500, 1000, 1500, and 2000 L/h.

Analysis of the error distribution shows that it ranges from ±1%. In addition, some values are more likely than others. This behaviour can be explained by the lack of resolution of the solid-state meters since the test bench under steady flow conditions is highly repetitive. Consequently, testing the meters in the laboratory under conditions like those found in the field implies an increase in the uncertainty of the test. This invites reflection on whether the resolution of solid-state meters can be modified according to the situation in which they operate (laboratory or field).

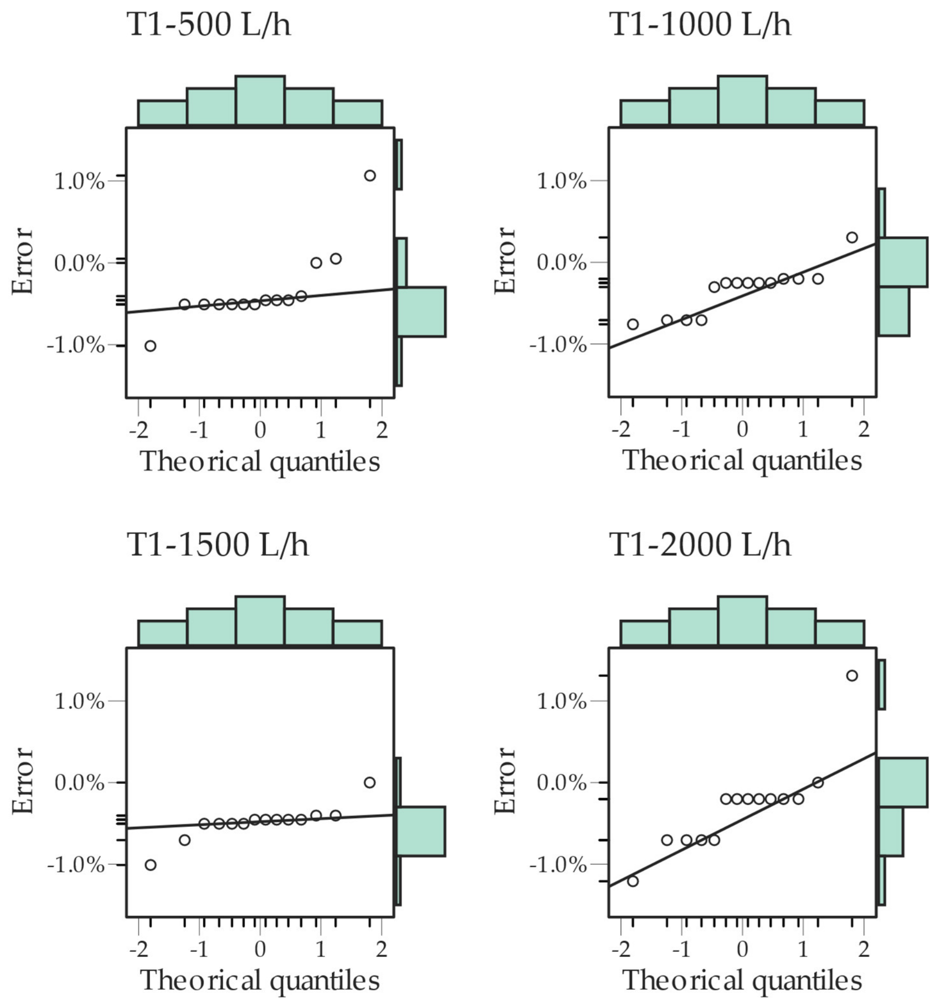

Figure S-1. Error obtained for the M0016 meter in test T1 (steady flow conditions) at flow rates of 500, 1000, 1500, and 2000 L/h that has been repeated 14 times.

2. *Number of repetitions conducted by test*

Table Table **S-1**, Table **S-2**, and Table **S-3** show the number of repetitions carried out per meter and test performed. From the information provided through these tables can be inferred how much data has been used to calculate an average error value or a box-whisker graph.

Table S-1. Number of repetitions conducted by test under steady flow conditions (T1). "Type of meter" and "Meter" are the first (*) and second (**) columns, respectively.

		T1										
		Flow (L/h)										
(*)	(**)	20	50	200	500	1000	1500	2000	2500	3000	4000	5000
M1	M0001	4	-	5	7	7	7	7	9	9	-	-
	M0008	4	-	5	7	7	7	7	9	9	-	-
	M0010	4	-	5	7	7	7	7	9	9	-	-
M2 (14)	M0023	-	-	3	3	-	-	3	-	-	-	-
	M0024	-	-	3	3	-	-	3	-	-	-	-
	M0025	-	-	3	3	-	-	3	-	-	-	-
	M0026	-	-	3	3	-	-	3	-	-	-	-
	M0029	-	-	3	3	-	-	7	-	-	-	-
M2 (17-18)	M0002	4	-	5	7	7	7	7	9	9	-	-
	M0007	4	-	5	7	7	7	7	9	9	-	-
	M0009	4	-	5	7	7	7	7	9	9	-	-
	M0022	-	-	3	3	-	-	3	-	-	-	-
	M0027	-	-	3	3	-	-	7	-	-	-	-
	M0028	-	-	3	3	-	-	7	-	-	-	-
	M0030	-	-	3	3	-	-	7	-	-	-	-
	M0031	-	-	3	3	-	-	7	-	-	-	-
M3	M0003	4	-	5	7	7	7	7	9	9	-	-
	M0005	4	-	5	7	7	7	7	9	9	-	-
M4	M0004	4	-	5	7	7	7	7	9	9	-	-
M6	M0011	-	5	6	7	7	7	7	7	9	-	-
	M0012	-	5	6	7	7	7	7	7	9	-	-
	M0013	-	5	6	7	7	7	7	7	9	-	-
	M0014	-	5	6	7	7	7	7	7	9	-	-
	M0015	-	5	6	7	7	7	7	7	9	-	-
M7	M0016	-	8	9	14	14	14	14	16	17	16	16
	M0017	-	4	5	7	7	7	7	9	9	9	9
M8	M0018	-	8	9	14	14	14	14	16	17	16	16
M9	M0019	-	4	5	7	7	7	7	9	9	9	9
	M0020	-	4	4	7	7	7	7	7	8	7	7
M10	M0021	-	4	4	7	7	7	7	7	8	7	7
	M0032	-	-	3	3	-	-	3	-	-	-	-
	M0033	-	-	3	3	-	-	3	-	-	-	-
	M0034	-	-	3	3	-	-	3	-	-	-	-
	M0035	-	-	3	3	-	-	3	-	-	-	-

Table S-2. Number of repetitions conducted by test under intermittent and constant flow conditions (T2, T3, T4, and T5). "Type of meter" and "Meter" are the first (*) and second (**) columns, respectively.

		T2			T3			T4					T5		
		Flow (L/h)													
(*)	(**)	200	500	2000	200	500	2000	200	500	1000	1500	2000	200	500	2000
M1	M0001	3	3	3	3	3	3	3	5	5	5	5	3	3	3
	M0008	3	3	3	3	3	3	3	5	5	5	5	3	3	3
	M0010	3	3	3	3	3	3	3	5	5	5	5	3	3	3
M2 (14)	M0023	-	-	-	3	3	3	3	3	-	-	3	3	3	3
	M0024	-	-	-	3	3	3	3	3	-	-	3	3	3	3
	M0025	-	-	-	3	3	3	3	3	-	-	3	3	3	3
	M0026	-	-	-	3	3	3	3	3	-	-	3	3	3	3
	M0029	-	-	-	3	3	3	3	3	-	-	3	3	3	3
M2 (17-18)	M0002	3	3	3	3	3	3	3	5	5	5	5	3	3	3
	M0007	3	3	3	3	3	3	3	5	5	5	5	3	3	3
	M0009	3	3	3	3	3	3	3	5	5	5	5	3	3	3
	M0022	-	-	-	3	3	3	3	3	-	-	3	3	3	3
	M0027	-	-	-	3	3	3	3	3	-	-	3	3	3	3
	M0028	-	-	-	3	3	3	3	3	-	-	3	3	3	3
	M0030	-	-	-	3	3	3	3	3	-	-	3	3	3	3
	M0031	-	-	-	3	3	3	3	3	-	-	3	3	3	3
M3	M0003	3	3	3	3	3	3	3	5	5	5	5	3	3	3
	M0005	3	3	3	3	3	3	3	5	5	5	5	3	3	3
M4	M0004	3	3	3	3	3	3	3	5	5	5	5	3	3	3
M6	M0011	3	3	3	3	3	3	3	3	-	-	3	3	3	3
	M0012	3	3	3	3	3	3	3	3	-	-	3	3	3	3
	M0013	3	3	3	3	3	3	3	3	-	-	3	3	3	3
	M0014	3	3	3	3	3	3	3	3	-	-	3	3	3	3
	M0015	3	3	3	3	3	3	3	3	-	-	3	3	3	3
M7	M0016	3	3	3	3	3	3	3	3	-	-	3	3	3	3
	M0017	3	3	3	3	3	3	3	3	-	-	3	3	3	3
M8	M0018	3	3	3	3	3	3	3	3	-	-	3	3	3	3
M9	M0019	3	3	3	3	3	3	3	3	-	-	3	3	3	3
	M0020	3	3	3	3	3	3	3	3	-	-	3	3	3	3
M10	M0021	3	3	3	3	3	3	3	3	-	-	3	3	3	3

Table S-3. Number of repetitions conducted by test under intermittent and variable flow conditions (T6 and T7). "Type of meter" and "Meter" are the first (*) and second (**) column, respectively.

		T6		T7		
		Flow profile				
**(*)**	(*)	**P1**	**P3**	**P1**	**P2**	**P3**
**M1**	**M0001**	3	4	4	3	4
	**M0008**	3	4	4	3	4
	**M0010**	3	4	4	3	4
**M2 (17-18)**	**M0002**	3	4	4	3	4
	**M0007**	3	4	4	3	4
	**M0009**	3	4	4	3	4
**M3**	**M0003**	3	4	4	3	4
	**M0005**	3	4	4	3	4
**M4**	**M0004**	3	4	4	3	4
**M6**	**M0011**	3	3	3	3	3
	**M0012**	3	3	3	3	3
	**M0013**	3	3	3	3	3
	**M0014**	3	3	3	3	3
	**M0015**	3	3	3	3	3
**M7**	**M0016**	3	3	3	3	3
	**M0017**	3	3	3	3	3
**M8**	**M0018**	3	3	3	3	3
**M9**	**M0019**	3	3	3	3	3
	**M0020**	3	3	3	3	3
**M10**	**M0021**	3	3	3	3	3

3. Hypothesis testing methods overview

Hypothesis testing is one of the foundations of inferential statistics [1]. In the present study, it is used for two purposes: i) contrast if there is equality of variances of two normally distributed populations; ii) comparison between the mean of two normally distributed populations whose variances are unknown. The hypothesis testing follows the next structure: The null hypothesis (the equality of means or variances of two populations in the case studied), which remains true unless there is strong evidence for the opposite, is contrasted with the alternative hypothesis (there is a difference between means or population variances), which is bilateral in the cases studied; then a decision rule is established to reject or accept the null hypothesis depending on the level of significance, which is equal to the probability of rejecting the null hypothesis being true (type I error) or, in other words, the probability of obtaining a false positive.

The level of significance should be as small as possible. However, a decrease in type I error leads to an increase in type II error (the probability of rejecting the alternative hypothesis when it is true or the probability of obtaining a false negative). Therefore, doubts about the veracity of the result obtained increase if the value taken is very small.

On the other hand, *effect size* and *power* are used to properly understand the results obtained from the hypothesis testing. The power is the complementary probability of the type II error, i.e. the probability of accepting an alternative hypothesis that is true (true positive). Therefore, the power does not affect the decision rule, but informs about its properties. For example, an increase in the number of meters tested leads to an increase in the power of the hypothesis testing for the same level of significance [2], which can be used to assess the return on investment in a larger sample. The effect size is the difference between the null and alternative hypothesis in units of the population standard deviation [1]. Thus, the importance of the difference found can be determined through the effect size, since a statistically significant difference is sometimes not meaningful from a technical point of view [2].

Finally, the p-value is used to examine the hypothesis testing. The p-value is the probability of obtaining a higher statistic than the observed if the null hypothesis was true [1]. Hence, it can be used to find the lowest significance level that defines when the null hypothesis can be rejected instead of using the pre-assigned values of significance [2]. In the present study, the significance level has been prefixed at 0.05, so if a $p\text{-value}<0.05$ is obtained, the null hypothesis will be rejected, based on a procedure that provides erroneous rejections with a 5% probability [3].

Regarding the specific methods used, the equality of variances between populations under comparison is contrasted through the F test [2], after verifying the normality of the data by means of the Shapiro-Wilk test [4] and the visualization of quantile-quantile plots with marginal histograms. Thereafter, the t-test [2] has been conducted to assess whether the performance of the M2 meters varies with age (independent samples) and flow conditions (paired samples). Finally, the effect size (*d* of Cohen) and the power of the test [5] have been calculated to analyse the results, which can be found in section 9 of the supplementary material.

R-statistics [6] is the software used to perform hypothesis testing. The external packages needed are: 1) *compute.es* [7] y *effsize* [8] to calculate *d* of Cohen in independent and paired samples, respectively; 2) *pwr* [9] to determine the power of test.

S-B. Supplementary material for (Section 3. Results)

4. Detection of typing errors in the database

Figure S-2 and Figure **S-3** show the graphs used in some of the database verifications. Both graphs refer to results obtained at the bench under intermittent flow conditions (described in section 2.1 of the research paper). In Figure S-2 it can be observed that the sum of the volume recorded by the reference meter of each line (MR004 and MR005) is within ±0.5% of the volume recorded by the reference meter at the bench output (MR003). At the same time, Figure S-3 verifies that 50%±5% of the total volume of the test passes through each line. The main conclusions derived from these graphs are: 1) the reference meters, which are volumetric type, perform very similarly under intermittent and steady flow conditions; 2) tests conducted on the bench under intermittent flow conditions are highly repetitive.

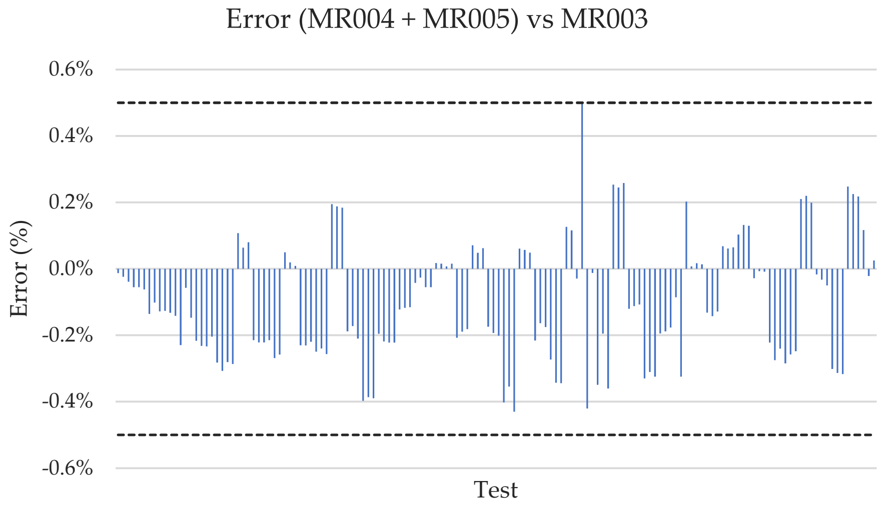

Figure S-2. Error of the sum of the volume recorded by the reference meters of each test line (Figure 2 of the research paper, MR004 and MR005) relative to the volume recorded by the reference meter at the output of the bench (Figure 2 of the research paper, MR003) used to conduct tests under intermittent flow conditions.

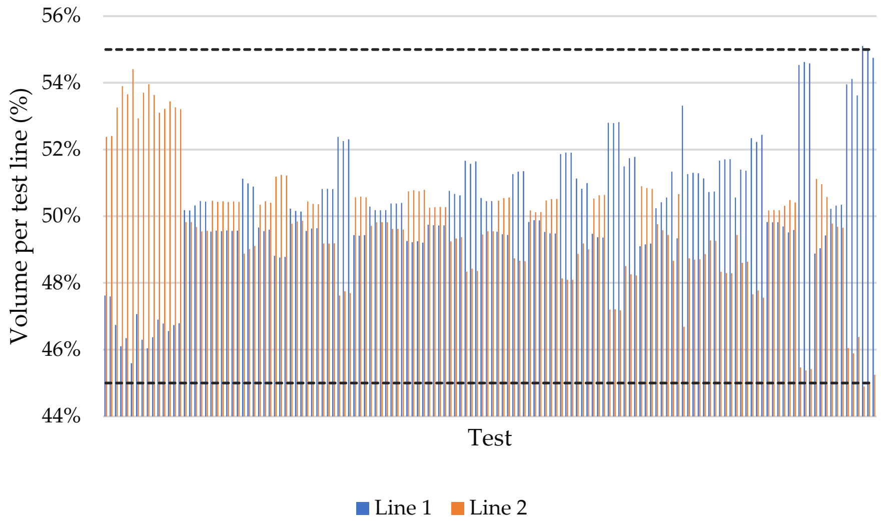

Figure S-3. Percentage of the total volume that passes through each line of the bench used to conduct tests under intermittent flow condition.

5. *Number of repetitions conducted by test*

Table S-4 and Table **S-5** show the mean and standard deviation of the error obtained per meter and test under steady flow conditions performed. These results are used as a reference for comparisons with the error obtained in tests under intermittent flow conditions.

Table S-4. Average error obtained per test under steady flow conditions (T1) and meter. "Type of meter" and "Meter" are the first (*) and second (**) column, respectively.

		**T1**										
		**Flow (L/h)**										
**(*)**	**(**)**	**20**	**50**	**200**	**500**	**1000**	**1500**	**2000**	**2500**	**3000**	**4000**	**5000**
**M1**	**M0001**	0.75%	-	0.31%	-0.47%	-0.22%	-0.32%	-0.12%	0.23%	0.41%	-	-
	**M0008**	0.69%	-	0.20%	0.66%	0.08%	0.24%	0.25%	0.31%	0.50%	-	-
	**M0010**	0.94%	-	-0.31%	-0.72%	-1.15%	-0.70%	-0.47%	-0.47%	-0.11%	-	-
**M2 (14)**	**M0023**	-	-	0.24%	0.34%	-	-	0.72%	-	-	-	-
	**M0024**	-	-	-0.78%	-0.85%	-	-	-0.45%	-	-	-	-
	**M0025**	-	-	0.07%	-0.34%	-	-	-0.45%	-	-	-	-
	**M0026**	-	-	0.07%	-0.17%	-	-	0.22%	-	-	-	-
	**M0029**	-	-	0.25%	-0.34%	-	-	0.14%	-	-	-	-
**M2 (17-18)**	**M0002**	0.23%	-	0.51%	0.18%	0.14%	-0.17%	-0.19%	0.46%	0.46%	-	-
	**M0007**	0.56%	-	-0.31%	0.08%	0.37%	0.45%	0.32%	0.47%	0.17%	-	-
	**M0009**	-0.46%	-	-0.31%	-0.07%	0.01%	0.24%	0.18%	0.31%	0.28%	-	-
	**M0022**	-	-	0.07%	0.00%	-	-	0.39%	-	-	-	-
	**M0027**	-	-	0.42%	-0.34%	-	-	-0.22%	-	-	-	-
	**M0028**	-	-	0.59%	0.51%	-	-	0.36%	-	-	-	-
	**M0030**	-	-	-0.09%	-0.51%	-	-	-0.14%	-	-	-	-
	**M0031**	-	-	-0.09%	-0.34%	-	-	-0.43%	-	-	-	-
**M3**	**M0003**	-0.16%	-	0.00%	-0.11%	0.14%	-0.39%	-0.48%	-0.10%	0.01%	-	-
	**M0005**	0.23%	-	-0.51%	-0.69%	-0.66%	-0.97%	-0.77%	0.18%	0.07%	-	-
**M4**	**M0004**	-1.06%	-	-0.61%	-0.76%	-0.66%	-0.68%	-0.55%	0.01%	0.18%	-	-
**M6**	**M0011**	-	-12.30%	1.06%	1.26%	1.53%	2.06%	2.12%	2.07%	1.92%	-	-
	**M0012**	-	0.14%	-0.23%	0.61%	1.87%	2.26%	2.39%	2.38%	2.43%	-	-
	**M0013**	-	-1.67%	-1.09%	-0.82%	0.33%	0.71%	0.81%	0.81%	0.71%	-	-
	**M0014**	-	1.44%	1.37%	1.08%	1.36%	1.39%	1.63%	1.70%	1.41%	-	-
	**M0015**	-	1.65%	0.70%	0.20%	0.30%	0.43%	0.87%	1.03%	0.95%	-	-
**M7**	**M0016**	-	-0.39%	-0.47%	-0.34%	-0.34%	-0.49%	-0.30%	0.01%	0.17%	-0.06%	-8.44%
	**M0017**	-	-0.59%	-0.12%	-0.50%	-0.33%	-0.53%	-0.27%	-0.17%	0.69%	-0.33%	-8.44%
**M8**	**M0018**	-	-0.01%	0.10%	-0.59%	-0.48%	-0.38%	-0.33%	-0.43%	-0.36%	-0.31%	-0.16%
**M9**	**M0019**	-	-1.13%	-0.48%	-1.05%	-0.64%	-0.53%	-0.46%	-0.61%	-0.81%	-1.04%	-1.30%
	**M0020**	-	-0.60%	-1.16%	-1.03%	-0.54%	-0.33%	-0.37%	-0.38%	-0.45%	-0.71%	-1.00%
**M10**	**M0021**	-	-0.71%	-0.65%	-0.75%	-0.64%	-0.73%	-0.75%	-0.92%	-0.73%	-0.79%	-0.74%
	**M0032**	-	-	-0.25%	-0.34%	-	-	-0.55%	-	-	-	-
	**M0033**	-	-	0.08%	-0.17%	-	-	-0.39%	-	-	-	-
	**M0034**	-	-	-0.08%	0.00%	-	-	-0.22%	-	-	-	-
	**M0035**	-	-	-0.08%	-0.17%	-	-	-0.55%	-	-	-	-

Table S-5. Standard deviation obtained per test under steady flow conditions (T1) and meter. "Type of meter" and "Meter" are the first (*) and second (**) column, respectively.

		**T1**										
		**Flow (L/h)**										
**(*)**	**(**)**	**20**	**50**	**200**	**500**	**1000**	**1500**	**2000**	**2500**	**3000**	**4000**	**5000**
**M1**	**M0001**	0.27%	-	0.43%	0.28%	0.29%	0.25%	0.36%	0.64%	0.64%	-	-
	**M0008**	0.25%	-	0.00%	0.24%	0.26%	0.26%	0.34%	0.28%	0.41%	-	-
	**M0010**	0.64%	-	0.00%	0.28%	0.23%	0.25%	0.27%	0.43%	0.36%	-	-
**M2 (14)**	**M0023**	-	-	0.03%	0.29%	-	-	0.32%	-	-	-	-
	**M0024**	-	-	0.03%	0.29%	-	-	0.74%	-	-	-	-
	**M0025**	-	-	0.28%	0.29%	-	-	0.79%	-	-	-	-
	**M0026**	-	-	0.28%	0.29%	-	-	0.28%	-	-	-	-
	**M0029**	-	-	0.01%	0.29%	-	-	0.24%	-	-	-	-
**M2 (17-18)**	**M0002**	0.23%	-	0.85%	0.38%	0.25%	0.51%	0.78%	0.91%	1.11%	-	-
	**M0007**	0.29%	-	0.00%	0.19%	0.25%	0.19%	0.65%	0.40%	0.42%	-	-
	**M0009**	0.66%	-	0.00%	0.20%	0.41%	0.26%	0.48%	0.27%	0.63%	-	-
	**M0022**	-	-	0.32%	0.00%	-	-	0.53%	-	-	-	-
	**M0027**	-	-	0.30%	0.29%	-	-	0.04%	-	-	-	-
	**M0028**	-	-	0.31%	0.00%	-	-	0.33%	-	-	-	-
	**M0030**	-	-	0.30%	0.00%	-	-	0.33%	-	-	-	-
	**M0031**	-	-	0.30%	0.29%	-	-	0.26%	-	-	-	-
**M3**	**M0003**	0.07%	-	0.28%	0.26%	0.25%	0.19%	0.47%	0.51%	0.61%	-	-
	**M0005**	0.27%	-	0.28%	0.27%	0.20%	0.30%	0.32%	0.58%	0.66%	-	-
**M4**	**M0004**	0.30%	-	0.28%	0.29%	0.20%	0.28%	0.22%	0.63%	0.80%	-	-
**M6**	**M0011**	-	0.78%	0.04%	0.10%	0.07%	0.05%	0.08%	0.02%	0.06%	-	-
	**M0012**	-	0.30%	0.08%	0.16%	0.10%	0.07%	0.03%	0.10%	0.05%	-	-
	**M0013**	-	0.39%	0.15%	0.10%	0.05%	0.06%	0.03%	0.07%	0.06%	-	-
	**M0014**	-	0.24%	0.09%	0.07%	0.05%	0.08%	0.06%	0.03%	0.19%	-	-
	**M0015**	-	0.27%	0.06%	0.03%	0.08%	0.02%	0.06%	0.06%	0.09%	-	-
**M7**	**M0016**	-	0.38%	0.41%	0.47%	0.29%	0.21%	0.57%	0.57%	0.89%	0.93%	0.75%
	**M0017**	-	0.25%	0.24%	0.29%	0.56%	0.30%	0.19%	0.24%	0.70%	1.03%	0.22%
**M8**	**M0018**	-	0.26%	0.24%	0.22%	0.25%	0.23%	0.22%	0.35%	0.30%	0.36%	0.33%
**M9**	**M0019**	-	0.24%	0.05%	0.31%	0.08%	0.06%	0.05%	0.04%	0.06%	0.07%	0.05%
	**M0020**	-	0.13%	0.44%	0.05%	0.08%	0.03%	0.11%	0.05%	0.02%	0.02%	0.02%
**M10**	**M0021**	-	0.42%	0.30%	0.26%	0.20%	0.27%	0.52%	0.47%	0.17%	0.38%	0.49%
	**M0032**	-	-	0.00%	0.29%	-	-	0.31%	-	-	-	-
	**M0033**	-	-	0.29%	0.29%	-	-	0.32%	-	-	-	-
	**M0034**	-	-	0.29%	0.51%	-	-	0.53%	-	-	-	-
	**M0035**	-	-	0.29%	0.29%	-	-	0.31%	-	-	-	-

6. Results of test under inttermitent flow conditions per meter

Figure S-4 includes the results of the intermittent flow tests (T2, T3, T4, T5, T6 and T7) obtained per meter. This is intended to complement Figures 13 and 14 in section 3.2 of the research paper, in which the same results are plotted by the type of meter.

a)

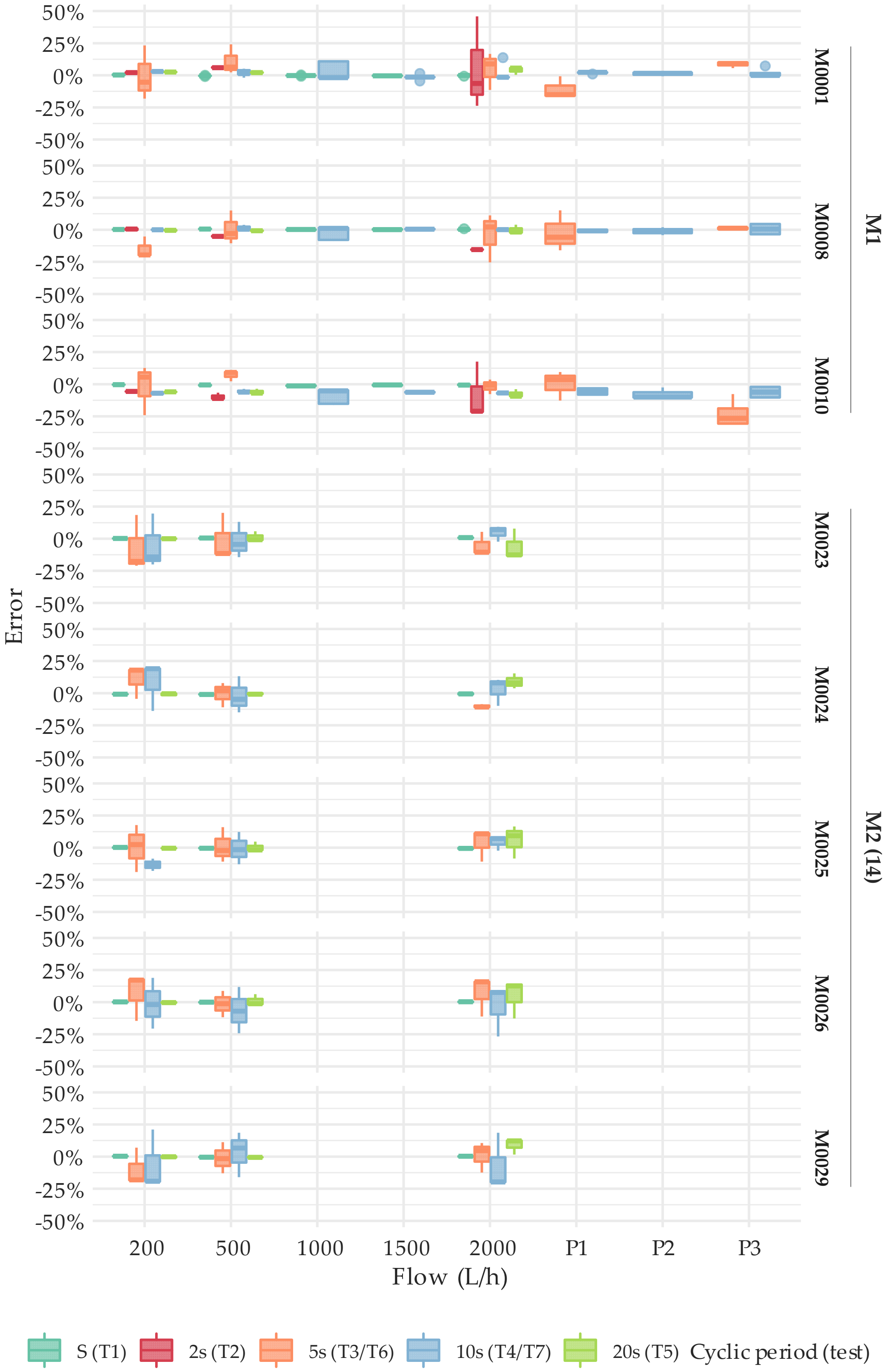

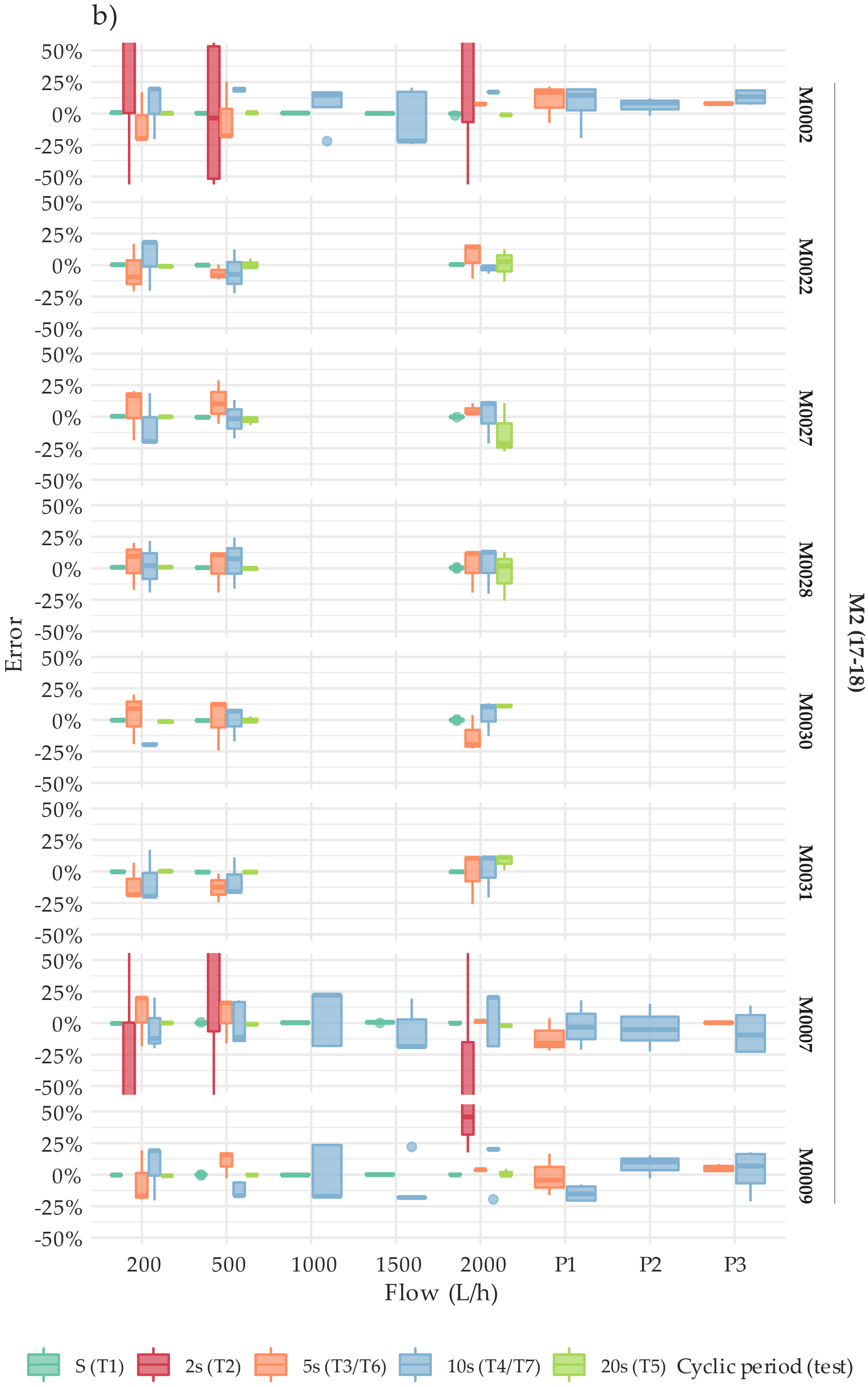
b)
50%
25%
0%
-25%
-50%
Error
M0002
M0022
M0027
M0028
M0030
M0031
M0007
M0009
M2 (17-18)
200
500
1000
1500
2000
P1
P2
P3
Flow (L/h)
S (T1)
2s (T2)
5s (T3/T6)
10s (T4/T7)
20s (T5)
Cyclic period (test)

c)

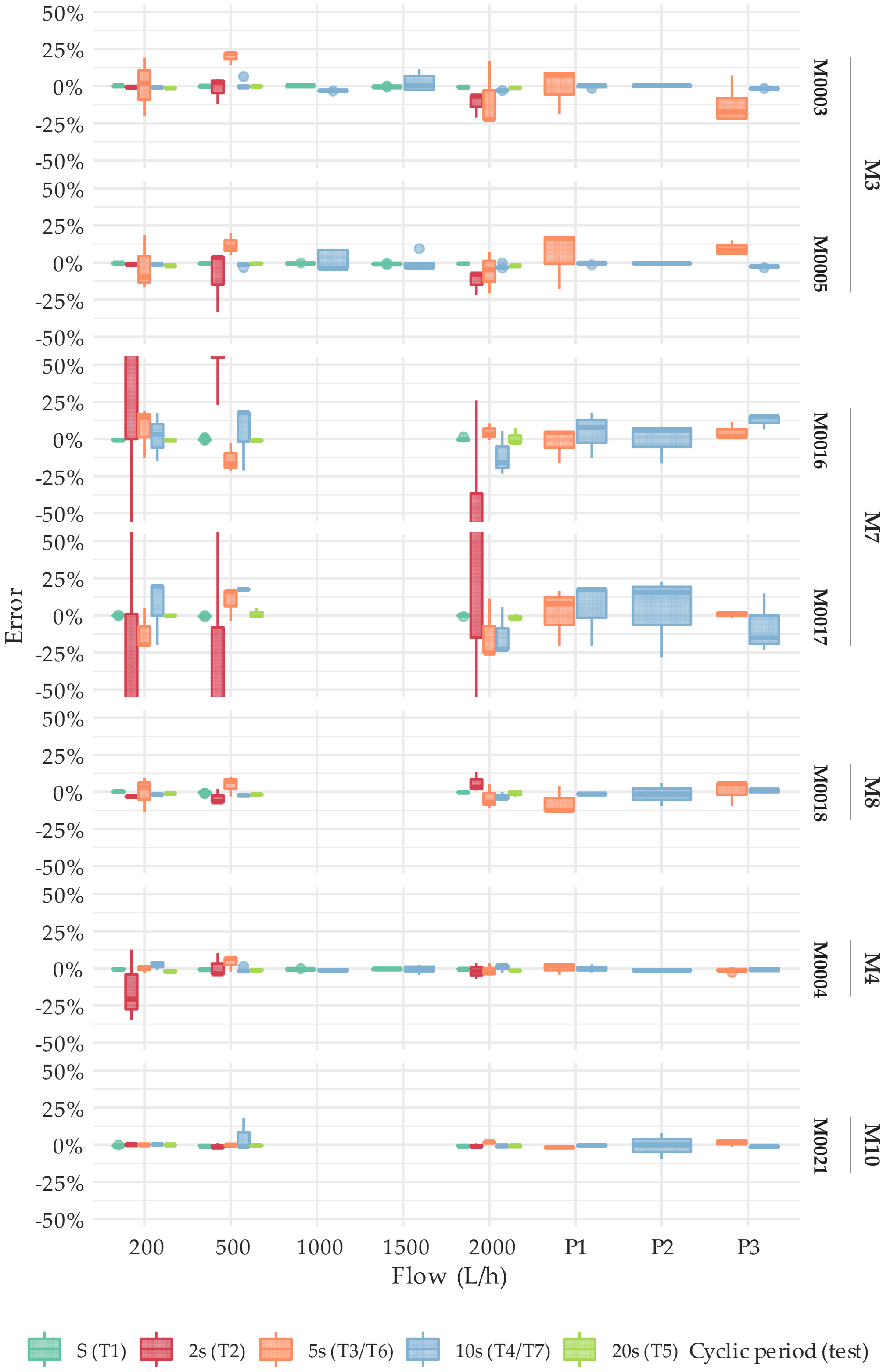
50%
25%
0%
-25%
-50%
Error
200
500
1000
1500
2000
P1
P2
P3
Flow (L/h)
M0003
M0005
M0016
M0017
M0018
M0004
M0021
M3
M7
M8
M4
M10
S (T1)
2s (T2)
5s (T3/T6)
10s (T4/T7)
20s (T5)
Cyclic period (test)

d)

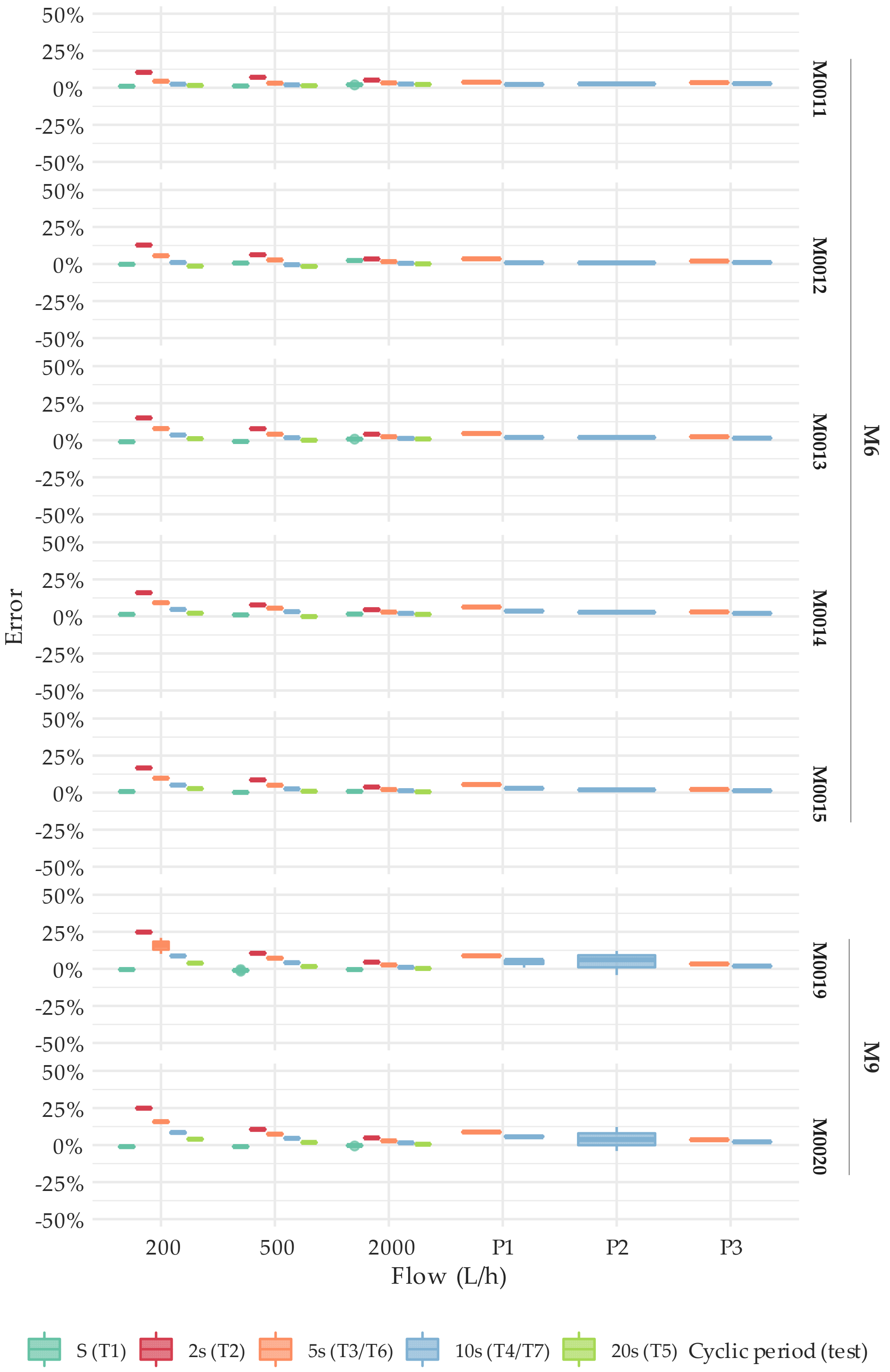

Figure S-4. Representation of the errors obtained in tests T2, T3, T4, T5, T6 and T7 by means of boxplots per meter, cyclic period, and test flow rate. Results in T1 (steady flow conditions) tests have been added as a reference.

7. Percentage of test results outside the error range of ±5%

According to ISO 4064-1 in all its versions since 1993, the maximum permissible error (MPE) of a class 2 water meter is set to ±5% in the lower range, between Q_1 and Q_2, and ±2% in the upper range, between Q_2 and Q_4. Taking as reference the largest MPE, this section describes the number of tests in which the error of the meters tested was greater than the MPE for the various test types considered. For specific test condition, if a high percentage of errors fall outside the permissible limits, an acceptable metrology will only be achieved if the errors between different tests are mutually compensated. This implies that measurement of individual water consumptions, under these conditions, is highly inaccurate, and an acceptable accuracy can only be achieved when a large number of consumptions are considered. Consequently, the actual accuracy of the meter mainly depends on the ability of the algorithms implemented by the manufacturer to reduce the bias caused by the sampling of the flow signal. At the present, none of the international regulations and standards related to water meters take into account this consideration in the test program.

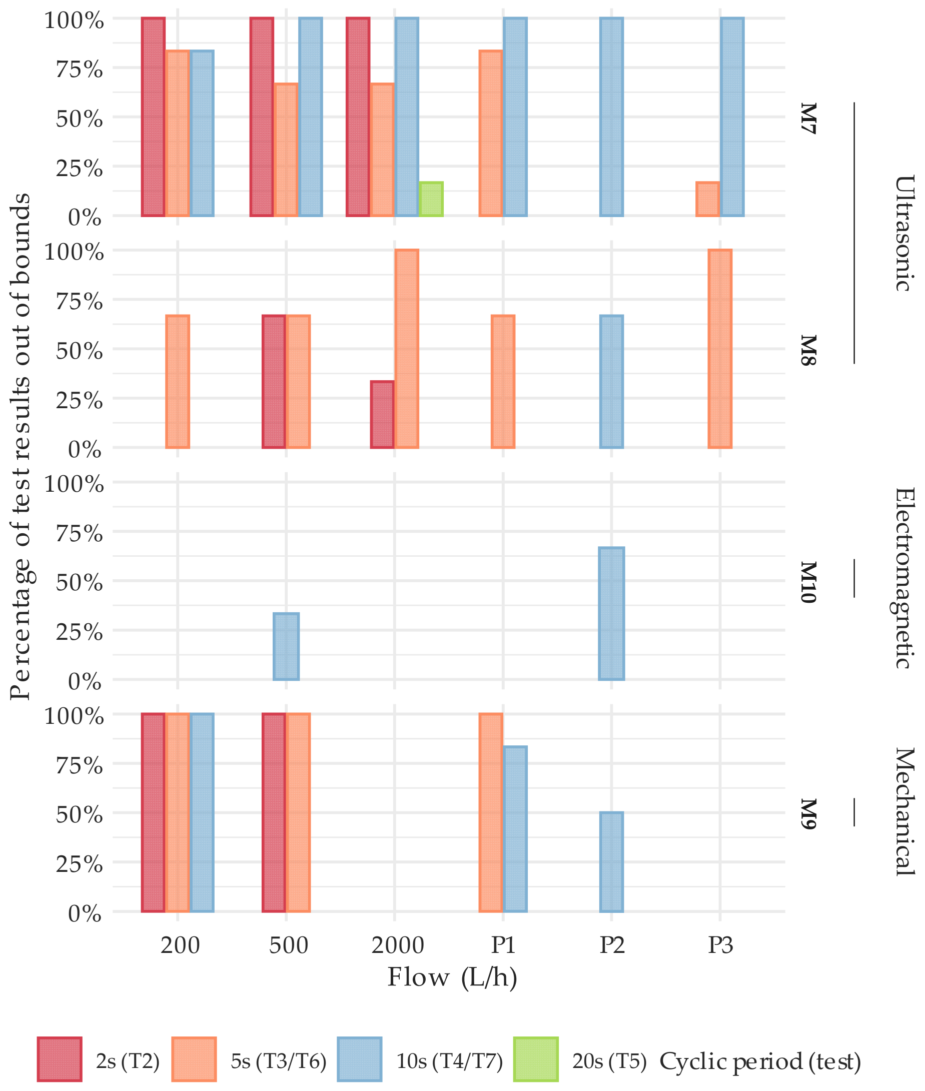

Figure S-5. Percentage of tests results outside the error range of ±5%. DN20.

As shown in Figure S-5 and Figure **S-6**, the percentage of tests in which measurement errors are larger than ±5% is exceptionally high, frequently reaching figures close to 100%, implying that the error of all tests conducted for that specific test type was greater than ±5%. In comparison with

ultrasonic meters, electromagnetic meters also showed large percentages of tests falling outside the MPE. However, from the results obtained is inferred that the ability of its algorithms to compensate positive and negative error is significantly better than the ones implemented in ultrasonic meters.

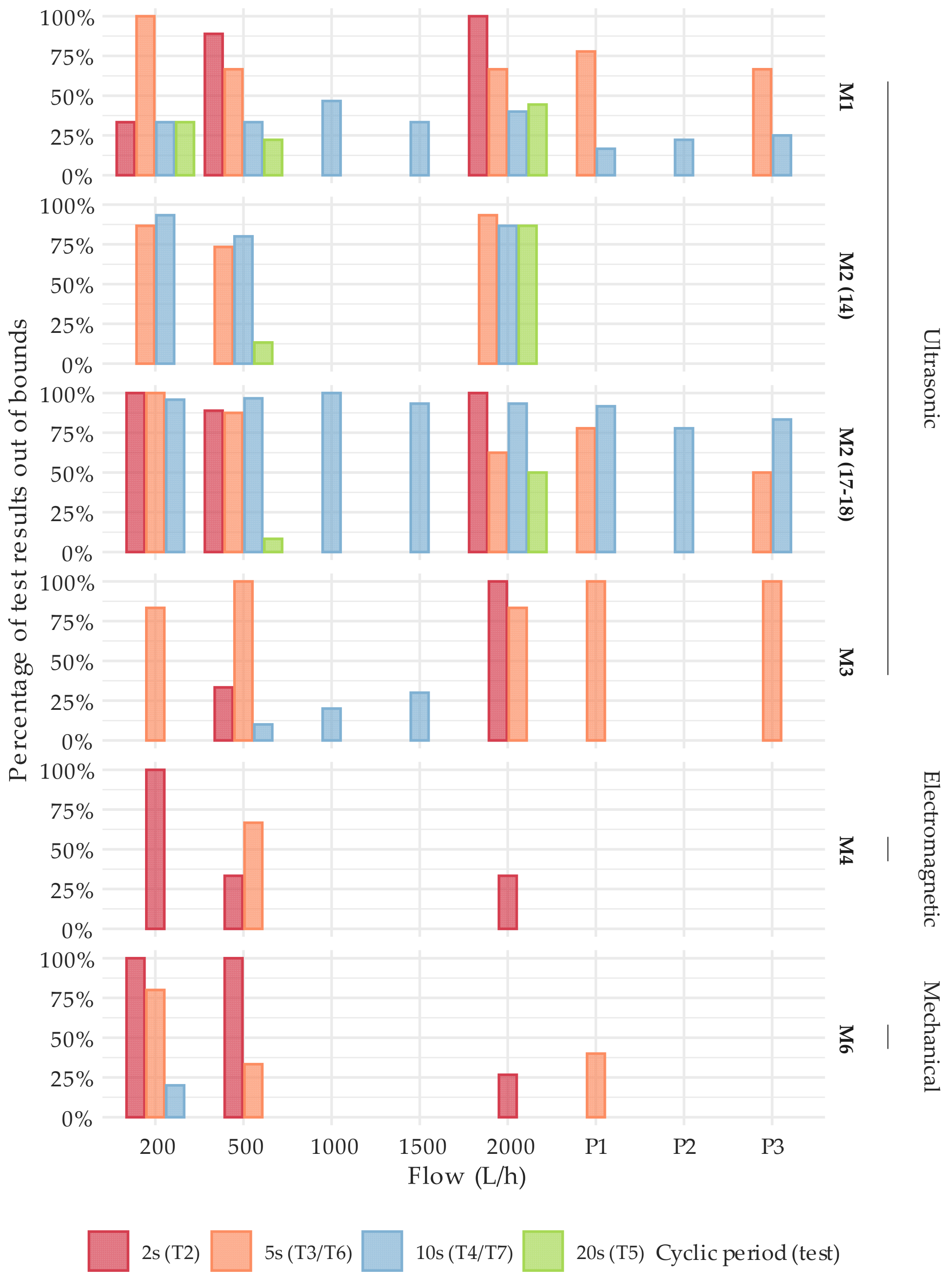

Figure S-6. Percentage of tests results outside the error range of ±5%. DN15.

8. Comparison of the calculated and measured error in tests T6 and T7

The objective of this section is to study the difference between the error measured on the test bench (Err_m) and the error calculated from the error curve and the consumption pattern (Err_c) when the meters are tested under intermittent and variable flow conditions. The results obtained in T6 and T7 test are used to conduct this analysis. The description of these tests can be found in section 2.3 of the research paper.

The value used as a measured error is the average of the errors obtained in the repetitions conducted per test (Table S-3). Regarding the calculated error, Figure S-7-a includes the error curve of each meter achieved in the T1 tests (steady flow conditions). The error at flows not covered by the test program has been calculated by linear interpolation. Figure S-7-b shows the demand patterns related to each flow profile tested at T6 and T7. The calculated error per meter is obtained applying the equation (1), where Err_i is the error obtained under steady flow conditions at the flow i, Pat_i is the percentage of consumption associated with the flow i and n the number of flows that compose a certain profile (P1, P2 or P3, as described in section 2.3 of the research paper).

$$Err_c = \sum_{i=1}^{n} Err_i * Pat_i \tag{1}$$

a)

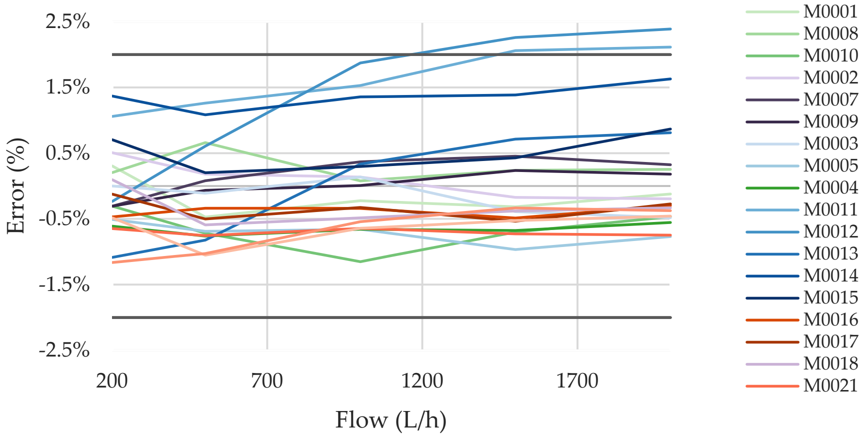

b)

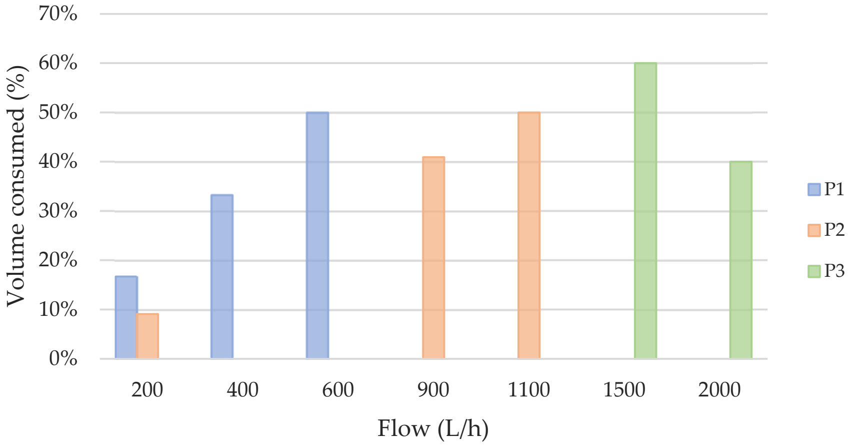

Figure S-7. a) Error curve per meter (based on results of T1 tests). b) Consumption pattern P1 (lower flow rate range), P2 (higher variability) and P3 (upper flow rate range).

Table S-6 and Table **S-7** summarise the results obtained for each meter per test conducted. In the case of electromagnetic and mechanical meters, the greatest differences between calculated and measured error are detected in test T6-P1 (consumption duration of 5 seconds and flow rate range between 200 and 600 L/h). In contrast, in the case of the ultrasonic meters, the response has been more variable. When the results are analysed by type of meter, M1, M3 and M8 generally perform worse at 5 second consumptions (T6), while for M2 and M7 the 10 second consumptions are more adverse (T7). On the other hand, the response obtained is more deficient for P1 (low flow range) or P3 (high flow range) profiles than P2, in which the flow variability is higher.

Table S-6. Error calculated (Err_c) and measured (Err_m) by meter in T6 and T7 test. Electromagnetic and mechanical meters.

Technology	Diameter	Type of meter	Meter	Type of test	Flow profile	Err_m	Err_c	$Err_m - Err_c$
EMF	DN15	M4	M0004	T6	P1	0.37%	-0.71%	*1.08%*
					P3	-1.49%	-0.63%	-0.86%
				T7	P1	-0.29%	-0.71%	0.41%
					P2	-1.06%	-0.66%	-0.40%
					P3	-0.75%	-0.63%	-0.12%
	DN20	M10	M0021	T6	P1	-1.82%	-0.71%	-1.11%
					P3	1.24%	-0.74%	*1.98%*
				T7	P1	-0.61%	-0.71%	0.10%
					P2	-0.59%	-0.66%	0.07%
					P3	-1.33%	-0.74%	-0.60%
M	DN15	M6	M0011	T6	P1	3.77%	1.23%	*2.54%*
					P3	3.55%	2.08%	1.47%
				T7	P1	2.27%	1.23%	1.04%
					P2	2.68%	1.52%	1.17%
					P3	2.83%	2.08%	0.75%
			M0012	T6	P1	3.43%	0.50%	*2.93%*
					P3	1.96%	2.31%	-0.35%
				T7	P1	0.89%	0.50%	0.40%
					P2	0.81%	1.62%	-0.81%
					P3	0.99%	2.31%	-1.32%
			M0013	T6	P1	4.62%	-0.78%	*5.40%*
					P3	2.35%	0.75%	1.60%
				T7	P1	1.91%	-0.78%	2.69%
					P2	1.86%	0.15%	1.71%
					P3	1.39%	0.75%	0.64%
			M0014	T6	P1	6.27%	1.19%	*5.08%*
					P3	3.03%	1.48%	1.55%
				T7	P1	3.70%	1.19%	2.51%
					P2	2.80%	1.34%	1.47%
					P3	2.06%	1.48%	0.58%
			M0015	T6	P1	5.45%	0.35%	*5.10%*
					P3	2.19%	0.60%	1.58%
				T7	P1	2.88%	0.35%	2.53%
					P2	1.89%	0.34%	1.55%
					P3	1.27%	0.60%	0.66%
	DN20	M9	M0019	T6	P1	8.83%	-0.85%	*9.68%*
					P3	3.32%	-0.50%	3.82%
				T7	P1	4.12%	-0.85%	4.97%
					P2	4.72%	-0.65%	5.37%
					P3	1.85%	-0.50%	2.35%
			M0020	T6	P1	8.70%	-1.02%	*9.71%*
					P3	3.59%	-0.35%	3.94%
				T7	P1	5.77%	-1.02%	6.79%
					P2	3.99%	-0.62%	4.61%
					P3	2.13%	-0.35%	2.48%

Table S-7. Error calculated (Err_c) and measured (Err_m) by meter in T6 and T7 test. Ultrasonic meters.

Technology	Diameter	Type of meter	Meter	Type of test	Flow profile	Err_m	Err_c	$Err_m - Err_c$
US	DN15	M1	M0001	T6	P1	-10.87%	-0.23%	*-10.64%*
					P3	8.50%	-0.24%	8.74%
				T7	P1	1.96%	-0.23%	2.19%
					P2	1.18%	-0.21%	1.39%
					P3	1.70%	-0.24%	1.94%
			M0008	T6	P1	-2.20%	0.48%	*-2.68%*
					P3	0.96%	0.24%	0.72%
				T7	P1	-0.78%	0.48%	-1.25%
					P2	-1.09%	0.15%	-1.24%
					P3	0.49%	0.24%	0.24%
			M0010	T6	P1	0.15%	-0.65%	0.80%
					P3	-22.94%	-0.61%	*-22.34%*
				T7	P1	-5.60%	-0.65%	-4.95%
					P2	-7.75%	-0.99%	-6.76%
					P3	-6.20%	-0.61%	-5.59%
		M2 (17-18)	M0002	T6	P1	10.17%	0.27%	9.91%
					P3	7.81%	-0.18%	7.99%
				T7	P1	7.24%	0.27%	6.97%
					P2	6.14%	0.14%	6.00%
					P3	12.97%	-0.18%	*13.15%*
			M0007	T6	P1	-11.27%	0.00%	*-11.27%*
					P3	0.27%	0.40%	-0.13%
				T7	P1	-2.34%	0.00%	-2.35%
					P2	-4.09%	0.29%	-4.38%
					P3	-6.95%	0.40%	-7.35%
			M0009	T6	P1	-1.31%	-0.12%	-1.19%
					P3	5.03%	0.21%	4.81%
				T7	P1	-14.72%	-0.12%	*-14.60%*
					P2	7.49%	0.00%	7.50%
					P3	2.56%	0.21%	2.35%
		M3	M0003	T6	P1	-0.49%	-0.05%	-0.43%
					P3	-12.36%	-0.43%	*-11.93%*
				T7	P1	-0.24%	-0.05%	-0.19%
					P2	0.09%	0.05%	0.04%
					P3	-1.49%	-0.43%	-1.06%
			M0005	T6	P1	5.45%	-0.64%	6.08%
					P3	9.37%	-0.89%	*10.26%*
				T7	P1	-0.68%	-0.64%	-0.04%
					P2	-0.49%	-0.68%	0.18%
					P3	-2.74%	-0.89%	-1.85%
	DN20	M7	M0016	T6	P1	-2.16%	-0.37%	-1.79%
					P3	4.94%	-0.41%	5.35%
				T7	P1	4.29%	-0.37%	4.66%
					P2	-0.70%	-0.37%	-0.34%
					P3	12.30%	-0.41%	*12.71%*
			M0017	T6	P1	1.24%	-0.38%	1.61%
					P3	0.48%	-0.43%	0.91%
				T7	P1	4.86%	-0.38%	5.24%
					P2	3.31%	-0.34%	3.65%
					P3	-7.77%	-0.43%	*-7.34%*
		M8	M0018	T6	P1	-7.01%	-0.39%	*-6.62%*
					P3	0.64%	-0.36%	1.00%
				T7	P1	-1.26%	-0.39%	-0.87%
					P2	-1.55%	-0.43%	-1.12%
					P3	0.55%	-0.36%	0.91%

Figure S-8 shows through box-whiskers the distribution of the difference between the measured and calculated error per meter. The main conclusions obtained are according to the research paper: i) under intermittent flow conditions, mechanical meters maintain repeatability, but tend to over-registration; ii) ultrasonic meters have a very variable response, observing problems of over and under registration; iii) the tested electromagnetic meters behave very similarly under steady and intermittent flow conditions. Thus, the electromagnetic meters tested measure correctly under intermittent and variable flow conditions, making them more competitive from a metrological point of view. However, the sample size should be increased to verify the results achieved. Regarding ultrasonic meters, the great variability of response and the notable difference observed between the measured and calculated error generates concerns about their ability to guarantee that all users will be treated equally. It can also happen that water services are adversely affected or favoured when the errors of different consumptions are not cancelled out. Unfortunately, these behaviours can only be identified through testing under intermittent flow conditions. For this reason, this type of tests should be included in the ISO 4064 and OIML R49 meter approval test program to protect both users and water utilities.

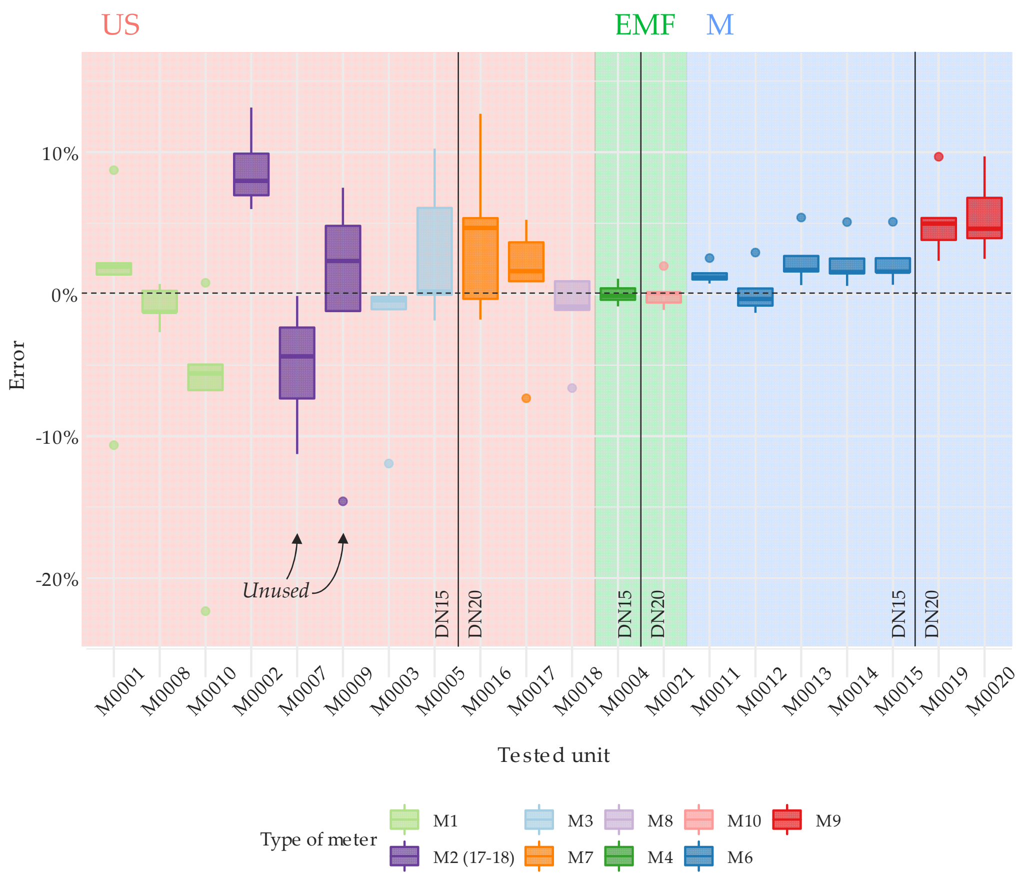

Figure S-8. Representation of the difference between the calculated and measured error per meter by means of boxplots, which are colored by type of meter.

9. Hypothesis testing results

The existence of statistically significant differences in the performance of M2 meters of different ages and under intermittent and steady flow conditions are analysed in this section. Other meter types examined in this study are not included in the analysis due to sample size limitations. In any case, the principles of the methodology followed here can be applied in the future to conduct comparisons such as the performance assessment between different meter types or to test the divergences between various batches from the same manufacturer.

The first analysis conducted studies the difference between the average error of two groups of meters: one manufactured in 2014 and in service for approximately 4 years and another manufactured between 2017 and 2018. The aim is to identify the influence on the metrology of the service period of a meter and whether its performance can be affected by changes implemented in the firmware over time. Two independent samples of 5 (2014) and 8 (2017-2018) meters are available, therefore the sample is unbalanced. Each sample has been tested under steady (T1) and intermittent (T3, T4 and T5) flow conditions at 200, 500 and 2000 L/h. The representative error of a given test is the mean of the errors obtained in each repetition carried out (the number of repetitions per test can be found in the Table S-1 and Table **S-2**).

The method used to solve the problem described is the independent or unpaired Student's t-test. The assumption of normality should be previously verified, as well as the homoscedasticity since it modifies the formulation of the test. The verifications conducted are: a) the Shapiro-Wilk test and the quantile-quantile plots with marginal histograms to check whether the normality of each defined group is satisfied; b) the F test to compare two variances and box-whisker plot to assess the existence of homoscedasticity in each comparison carried out. On the other hand, the significance level has been set at 0.05 and the effect size and power of the test are used to interpret the results. Finally, the optimal sample size to obtain appropriate values of the test power (80%) has been calculated, given an effect size for balanced experimental designs.

Table S-8 summarises the measures of position (mean and median), dispersion (standard deviation) and shape (skewness and kurtosis) used to describe the distribution of error in each group defined by the test type (e.g. T1 at 200 L/h or T5 at 2000 L/h) and age of the meter (M2 manufactured in 2014 and 2017-2018). For each group, the difference between the mean and median is usually around ±0.5% and does not exceed ±2.5%. The standard deviation of the tests under steady flow (T1) is considerably lower than the standard deviation of the tests under intermittent flow (T3, T4 and T5), with the exception of the T5 tests at medium flows (200 and 500 L/h). On the other hand, the coefficient of skewness takes values of ±0.5 in most cases. However, negative values of -1.6 are reached. Regarding the kurtosis, in general the results obtained are close to 3 (value associated to the normal distribution), but a trend towards leptokurtic and platykurtic distribution is observed in some cases (e.g. *M2 (17-18)/T5/500 L/h* and *M2 (14)/T5/500 L/h*, respectively).

Nevertheless, as the available sample is small, the conclusions drawn from these measures should be interpreted with caution. For this reason, the Shapiro-Wilk test and q-q plots have been used to decide on the normality of the error distribution in the analysed samples, as mentioned before. The null hypothesis (the sample is normally distributed) of the Shapiro-Wilk test is accepted in all cases, except in group *M2 (17-18)/T5/500 L/h*. Figure S-9 and Figure **S-10** show the q-q plots for meters M2 (14) and M2 (17-18), respectively, where it can be seen that most of the error values are above the line that would follow a normal distribution. The graph for group *M2 (17-18)/T5/500 L/h* (Figure S-9) reveals that only one meter (M0027) is outside the line. Consequently, the distribution of the error observed in the M2 meters tested under different types of test is close to a normal distribution. This conclusion allows the use of the parametric statistics tools and indicates that there is no bias in the metrological behaviour of M2 meters.

Table S-8. Descriptive statistics and results of the Shapiro-Wilk normality test (null hypothesis: the sample is normally distributed) of each defined group to compare the performance of M2 type meters of different ages.

Type of test	Flow (L/h)	Type of meter	Num. of units	Mean	sd	Median	Skewness	Kurtosis	Shapiro-Wilk test (Normality) W	Shapiro-Wilk test (Normality) p-value
T1	200	M2-(14)	5	-0.03%	0.43%	0.07%	-1.35	3.05	0.72	*0.014*
		M2-(17/18)	8	0.10%	0.36%	-0.01%	0.24	1.47	0.88	0.200
	500	M2-(14)	5	-0.27%	0.42%	-0.34%	0.13	2.38	0.95	0.740
		M2-(17/18)	8	-0.06%	0.33%	-0.03%	0.29	2.22	0.96	0.852
	2000	M2-(14)	5	0.04%	0.50%	0.14%	0.22	1.71	0.89	0.382
		M2-(17/18)	8	0.03%	0.32%	0.02%	-0.13	1.46	0.88	0.209
T3	200	M2-(14)	5	0.23%	8.69%	0.36%	0.01	1.44	0.94	0.696
		M2-(17/18)	8	-0.91%	6.80%	-0.56%	-0.09	1.36	0.89	0.260
	500	M2-(14)	5	-0.60%	0.94%	-1.05%	1.07	2.62	0.83	0.146
		M2-(17/18)	8	0.74%	8.22%	0.90%	-0.23	2.02	0.96	0.845
	2000	M2-(14)	5	-0.76%	6.88%	0.97%	-0.32	1.65	0.96	0.779
		M2-(17/18)	8	1.46%	6.53%	2.49%	-1.38	4.02	0.84	0.076
T4	200	M2-(14)	5	-3.44%	7.85%	-4.93%	0.34	2.30	0.97	0.855
		M2-(17/18)	8	-2.32%	8.85%	-1.25%	-0.73	2.54	0.89	0.228
	500	M2-(14)	5	-1.63%	3.43%	-1.85%	-0.05	2.39	0.96	0.776
		M2-(17/18)	8	-0.61%	9.48%	-1.46%	0.99	3.42	0.91	0.351
	2000	M2-(14)	5	0.32%	5.25%	2.86%	-0.54	1.58	0.85	0.190
		M2-(17/18)	8	4.76%	6.65%	2.83%	0.83	2.47	0.90	0.270
T5	200	M2-(14)	5	-0.39%	0.36%	-0.36%	-0.38	2.41	0.96	0.792
		M2-(17/18)	8	-0.35%	0.78%	-0.28%	-0.09	1.97	0.98	0.978
	500	M2-(14)	5	0.36%	0.79%	0.34%	0.06	1.32	0.89	0.381
		M2-(17/18)	8	-0.48%	1.10%	-0.29%	-1.60	4.73	0.78	*0.018*
	2000	M2-(14)	5	4.48%	6.21%	5.77%	-1.19	2.83	0.79	0.070
		M2-(17/18)	8	0.19%	7.34%	-0.20%	-0.14	2.67	0.95	0.742

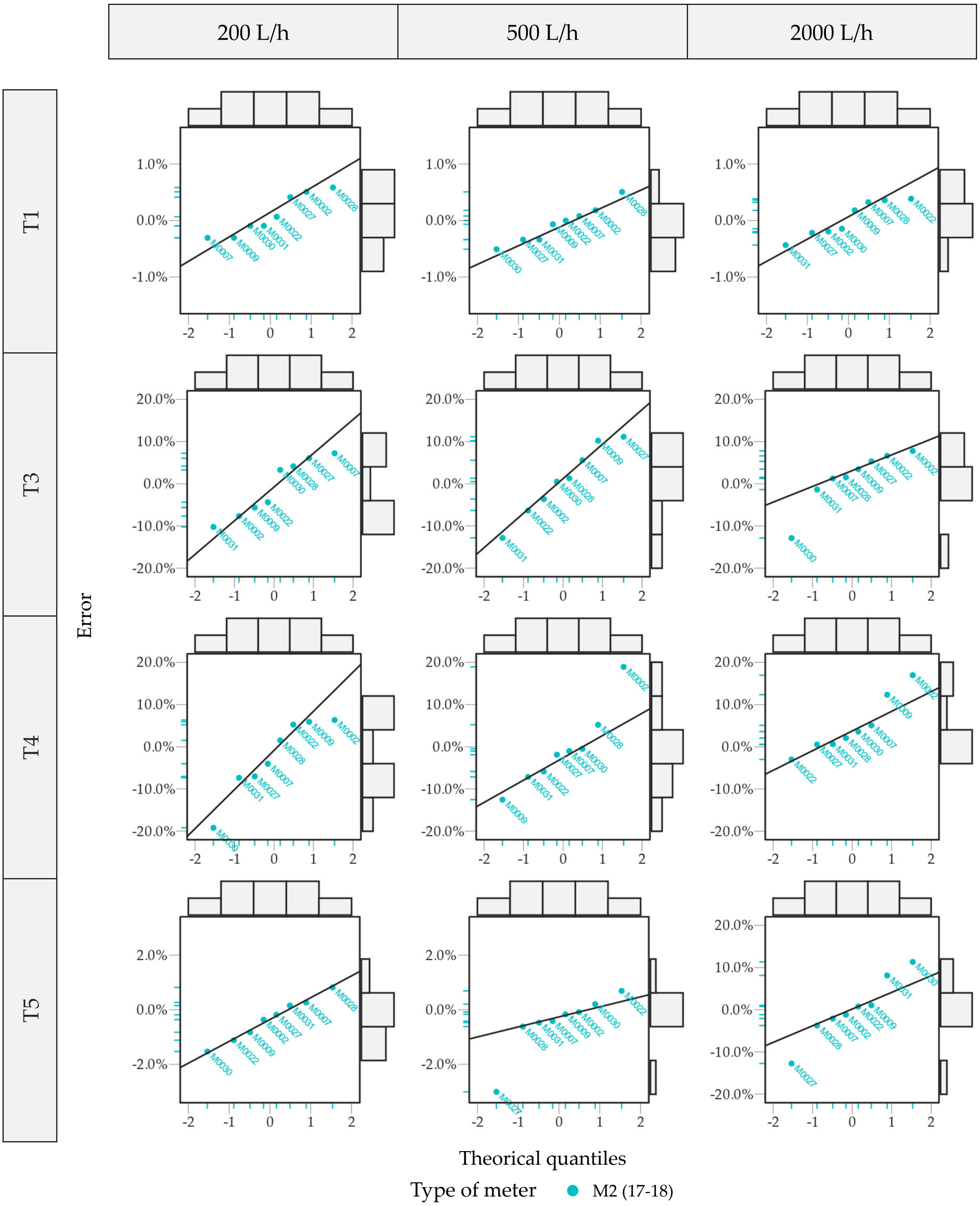

Figure S-9. Quantile-quantile plot with marginal histograms to assess graphically the normality of the error distribution of the M2 (17-18) meters obtained in different tests

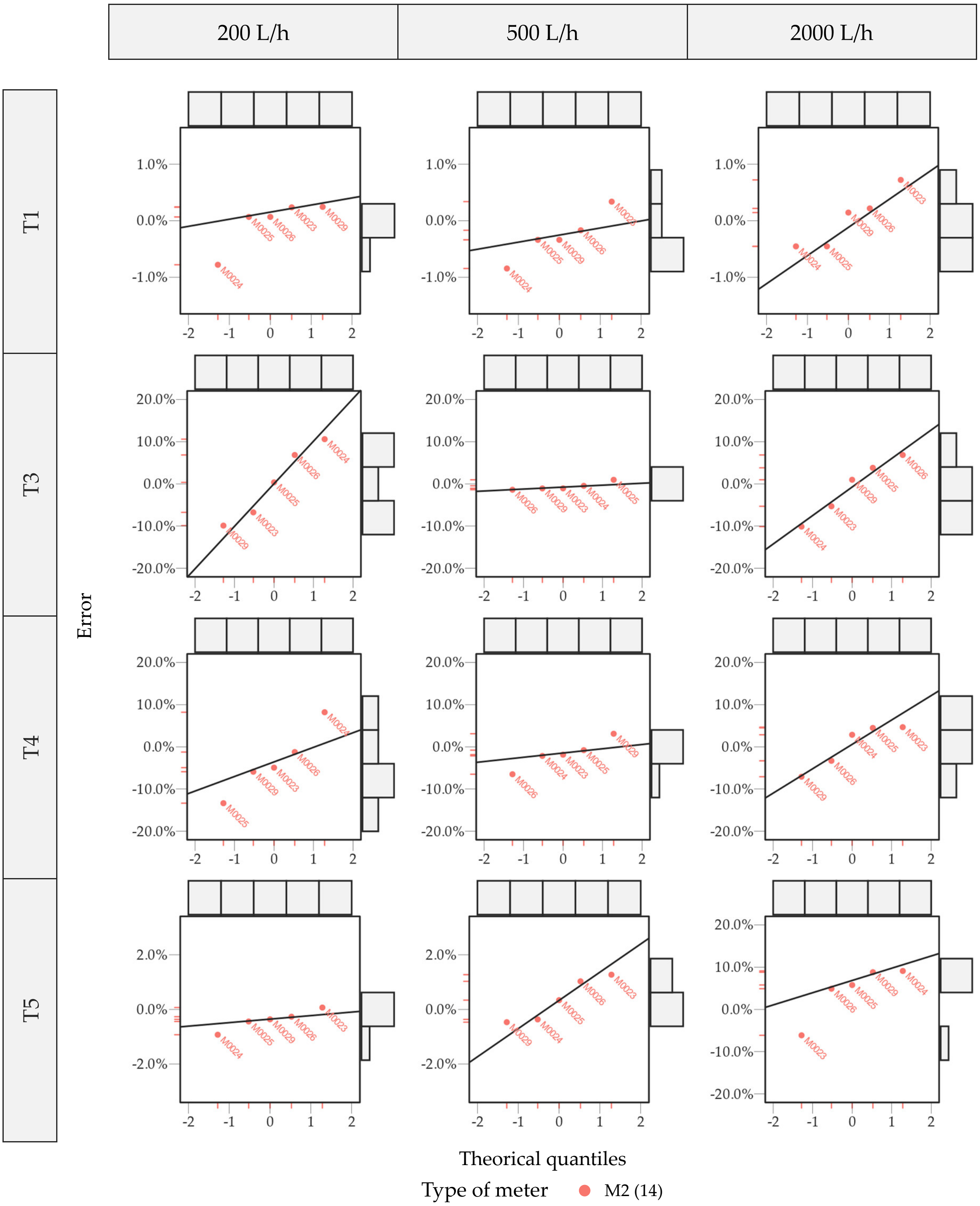

Figure S-10. Quantile-quantile plot with marginal histograms to assess graphically the normality of the error distribution of the M2 (14) meters obtained in different tests.

Table S-9 summarizes the results obtained in the t and F test. The conclusion is that the behavioural differences between the two age groups defined are statistically insignificant. Therefore, it can be stated that the changes implemented in the firmware of the M2 meters between 2014 and 2017-2018 and the service period of the meters manufactured in 2014 have not affected its metrology. Regarding homoscedasticity, only significant differences have been detected in the comparison of variances conducted in the T3-500 L/h case. This result is underlined by the boxplot shown in Figure S-11.

Table S-9. Results of the hypothesis testing conducted to assess the difference between the performance of M2 meters manufactured in 2014 and 2017-2018.

			F test (Homocedasticity)	t test				
**Hypothesis testing**	**Type of test**	**Flow (L/h)**	**p-value**	**t**	**df**	**p-value**	**Cohen's d**	**Power**
M2 (14) / M2 (17-18)	T1	200	0.648	-0.590	11	0.567	-0.340	0.085
		500	0.522	-1.007	11	0.336	-0.570	0.150
		2000	0.278	0.012	11	0.990	0.010	0.050
	T3	200	0.537	0.264	11	0.796	0.150	0.057
		500	*0.001*	-0.457	7.29	0.661	-0.260	0.070
		2000	0.843	-0.584	11	0.571	-0.330	0.083
	T4	200	0.862	-0.230	11	0.822	-0.130	0.055
		500	0.068	-0.230	11	0.822	-0.130	0.055
		2000	0.679	-1.260	11	0.234	-0.720	0.211
	T5	200	0.154	-0.104	11	0.919	-0.060	0.051
		500	0.539	1.482	11	0.166	0.840	0.270
		2000	0.785	1.083	11	0.302	0.620	0.169

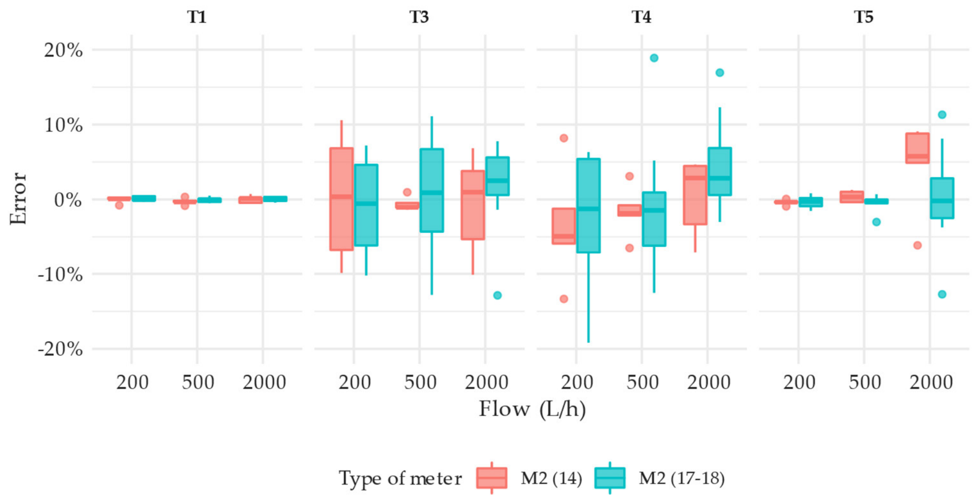

Figure S-11. Boxplots to compare the distribution of the error among the age groups.

The effect sizes are generally smaller than 0.2. Hence the population averages difference is relatively small compared to the variability within groups. In only four comparisons, this effect has exceeded 0.5. This result reinforces the conclusion that the differences found are negligible. On the other hand, the power of the test is generally low. For effect sizes as small as those detected, a very large sample size would be necessary to reach power values around 0.8 (Figure S-12). Since the aim is to identify mainly those cases where the effect size is large (>0.8), the optimal sample size for the hypothesis testing proposed is 25 meters per group (Figure S-12). Consequently, the conclusions are considered preliminary until the number of tested units can be increased.

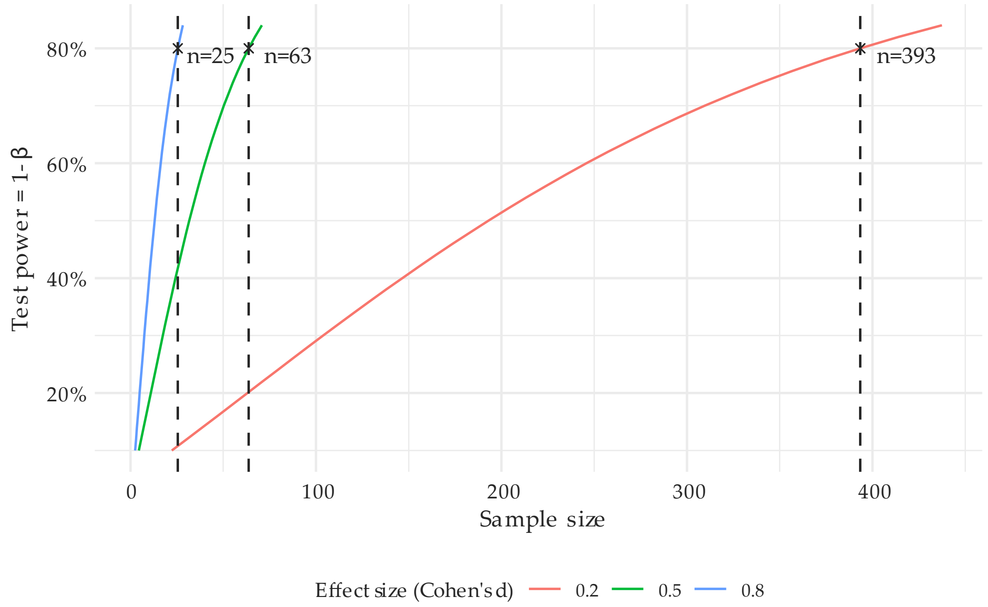

Figure S-12. Two independent sample t test power calculations. Two sided tails and alpha of 0.05. *n* is de optimal sample size (number of units in each group for a balanced experiment design).

The second analysis studies the differences in the average behaviour of M2 meters tested under steady and intermittent flow conditions. In this case, the hypothesis testing conducted is on dependent or paired samples. The control group is composed of the average errors obtained in the tests under steady flow conditions, and the number of units available per group is 13.

Table S-10. Descriptive statistics and results of the Shapiro-Wilk normality test (null hypothesis: the sample is normally distributed) of each group established to compare the performance of M2 meters under steady and intermittent flow conditions.

								Shapiro-Wilk test (Normality)
**Type of test**	**Flow (L/h)**	**Units**	**Mean**	**sd**	**Median**	**Skewness**	**Kurtosis**	**p-value**
T1	200	13	0.05%	0.37%	0.07%	-0.56	2.97	0.695
	500	13	-0.14%	0.37%	-0.17%	0.00	2.56	0.943
	2000	13	0.03%	0.38%	0.14%	0.12	1.93	0.339
T3	200	13	-0.47%	7.24%	0.36%	0.01	1.50	0.214
	500	13	0.22%	6.34%	-0.46%	-0.04	3.11	0.461
	2000	13	0.60%	6.47%	1.53%	-0.93	2.74	0.092
T4	200	13	-2.75%	8.16%	-4.03%	-0.41	2.39	0.557
	500	13	-1.00%	7.52%	-1.85%	1.25	5.06	0.061
	2000	13	3.05%	6.33%	2.86%	0.66	3.27	0.418
T5	200	13	-0.36%	0.63%	-0.36%	-0.06	2.61	0.997
	500	13	-0.16%	1.05%	-0.17%	-1.35	5.43	*0.026*
	2000	13	1.84%	7.00%	1.05%	-0.50	2.46	0.673

Table S-10 and Figure S-13 are similar to those associated with the first hypothesis testing proposed. The same conclusion is reached as in the first analysis conducted: the distribution of error

for each group is near to a normal distribution. Therefore, the assumption of normality that authorizes the use of the t and F test is satisfied.

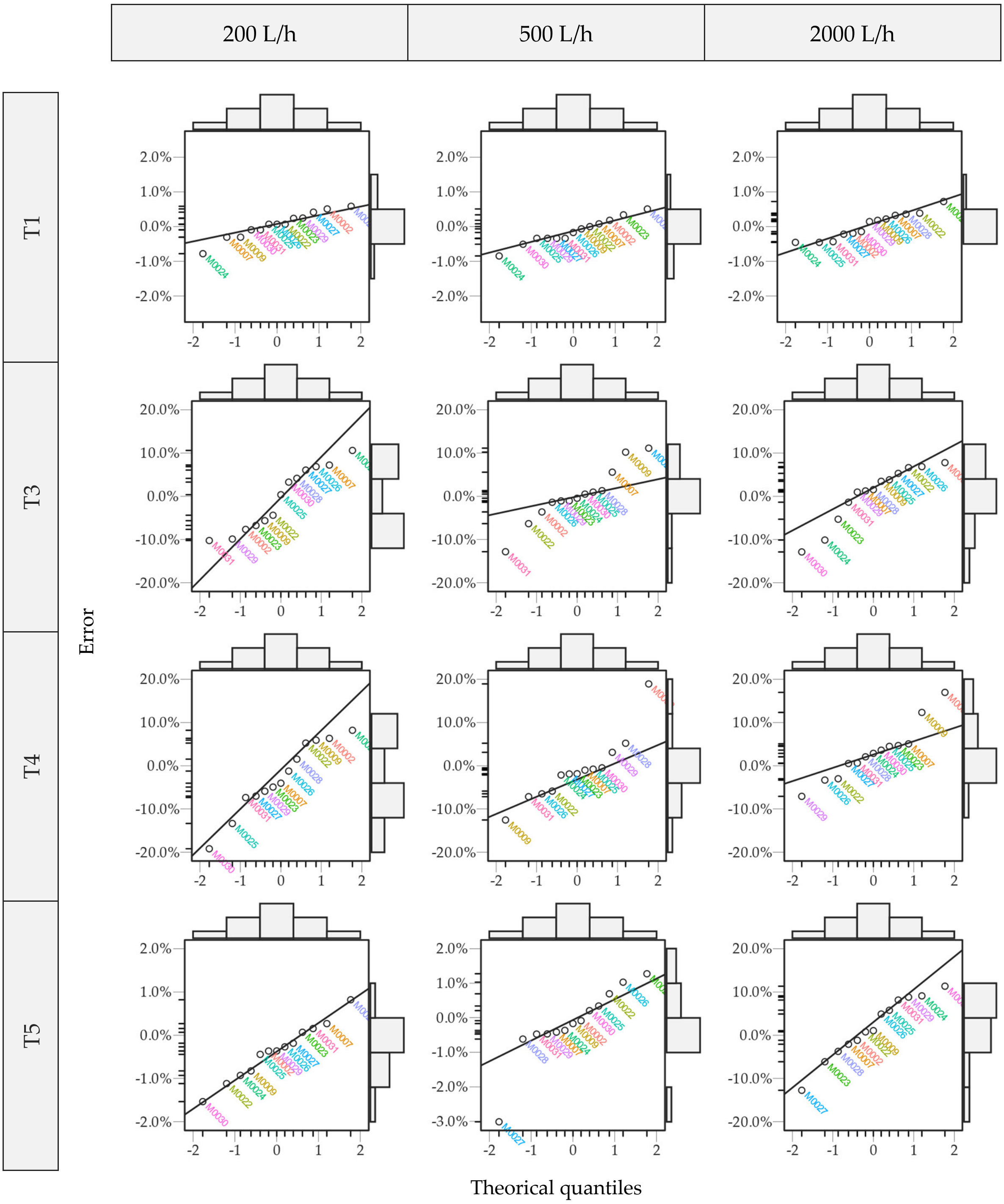

Figure S-13. Quantile-quantile plot with marginal histograms to graphically evaluate the normality of the error distribution of the M2 meters obtained in different tests.

Table S-11 summarizes the results obtained in the last hypothesis testing. Respecting the homoscedasticity, F test has returned a p-value lower than 0.05 in all cases, except for the comparison *T1-T5/200 L/h*. This result can be visually verified in Figure S-14, where the error distribution in T1 tests (steady flow conditions) has a notably lower dispersion than the dispersion obtained in the tests under intermittent flow conditions.

Despite the proposed intermittent flow tests force the meters to extreme operating conditions, the fact that this type of meter shows such a scattered behaviour generates uncertainty about its correct performance in the field. Although the average error of a M2 meter park is within the limits set by the standard and does not show over or under registration bias, it could be benefiting some users over others, or the water utilities could be adversely affected if the errors are not cancelled out.

Table S-11. Results of the hypothesis testing conducted to assess the difference between the performance of M2 meters under steady and intermittent flow conditions.

		F test (Homocedasticity)	t test				
**Hypothesis testing**	**Flow (L/h)**	**p-value**	**t**	**df**	**p-value**	**Cohen's d**	**Power**
T1 / T3	200	*3.335E-13*	-0.253	12	0.805	-0.114	0.067
	500	*1.274E-12*	0.206	12	0.840	0.080	0.058
	2000	*1.337E-12*	0.319	12	0.755	0.116	0.067
T1 / T4	200	*8.038E-14*	-1.229	12	0.243	-0.513	0.398
	500	*1.648E-13*	-0.418	12	0.683	-0.138	0.074
	2000	*1.755E-12*	1.704	12	0.114	0.711	0.654
T1 / T5	200	8.468E-02	-2.591	12	*0.024*	-0.756	0.706
	500	*9.492E-04*	-0.063	12	0.951	-0.022	0.051
	2000	*5.262E-13*	0.911	12	0.380	0.426	0.293

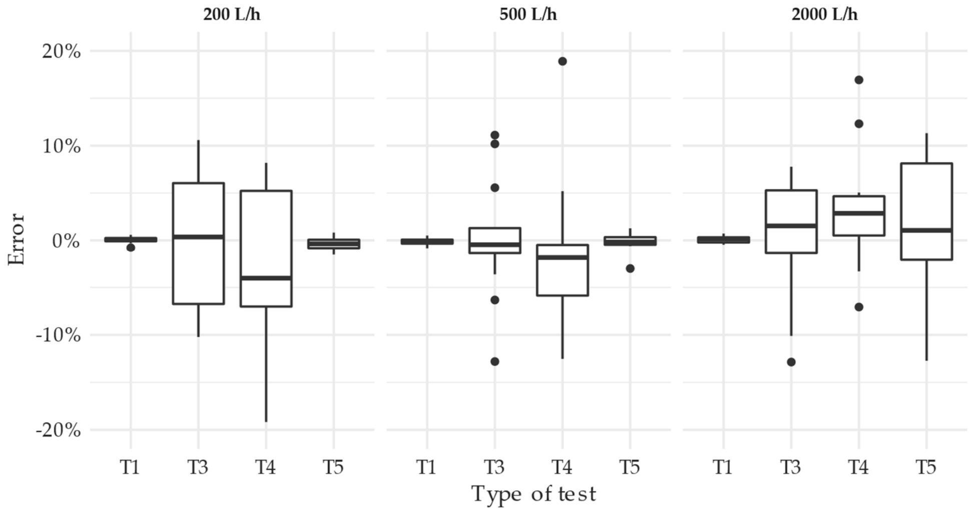

Figure S-14. Comparison of error distribution of M2 meters obtained in tests under continuous and intermittent flow conditions through boxplots.

Relating to the result of the t-test, *T1-T5/200 L/h* is the only case in which a statistically significant difference in the average error has been detected. This case has an associated effect size of -0.756, which in absolute value is equal to an error difference of 0.41%. This result invites two reflections: 1) it is necessary to expand the definition of significance [2], taking into account the order of magnitude of the effect size, since there are occasions in which the t-test detects a significant difference in the average error that is negligible from a technical point of view, as it occurs in the *T1-T5/200 L/h* case; 2) the effect size detected is directly related to the dispersion of the variable considered; therefore, when the variability observed in error is small, the effect size to be detected reaches high values (>0.8) and, consequently, the number of meters per group can be smaller without affecting the power of the

test. In contrast, when the variable dispersion is greater, the effect size will decrease and the number of units per group should be greater to reach appropriate power values (Figure S-15).

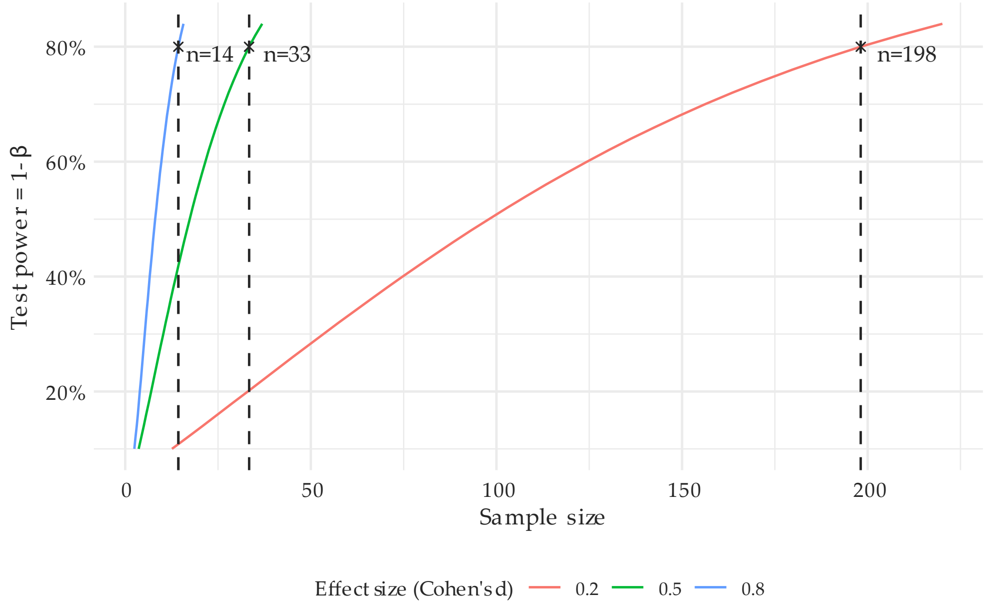

Figure S-15. Two paired sample t test power calculation. Two sided tails and alpha of 0.05. *n* is de optimal sample size (number of units in each group for a balanced experiment design).

References

1. Demidenko, E. Chapter 7: Hypothesis testing and confidence intervals. In *Advanced Statistics with Applications in R*; Wiley: Hoboken, NJ, US, 2020 ISBN 9781118387986.
2. Newbold, P.; Carlson, W.; Thorne, B. Chapter 9 (Hypothesis Testing: Single Population) and Chapter 10 (Hypothesis Testing: Additional Topics). In *Statistics for business and economics*; Carlson, W.L., Thorne, B.M., Eds.; Pearson Education International: Upper Saddle River [etc.], 2010 ISBN 9780135072486.
3. Rasch, D.; Schott, D. Chapter 3: Statistical tests and confidence estimations. In *Mathematical statistics*; Schott, D., Ed.; 2018 ISBN 1-119-38526-1.
4. Shapiro, S.S.; Wilk, M.B. An Analysis of Variance Test for Normality (Complete Samples). *Biometrika* **1965**, *52*, 591, doi:10.2307/2333709.
5. Cohen, J. Statistical power analysis for the behavioral sciences 1988.
6. Team, R.C. R: A language and environment for statistical computing 2019.
7. Del Re, A.C. compute.es: Compute Effect Sizes 2013.
8. Torchiano, M. effsize: Efficient Effect Size Computation 2020.
9. Champely, S. pwr: Basic Functions for Power Analysis 2020.
